# The basic leucine zipper domain (bZIP) transcription factor *BbYap1* promotes evasion of host humoral immunity and regulates lipid homeostasis contributing to fungal virulence in *Beauveria bassiana*

**DOI:** 10.1128/msphere.00351-24

**Published:** 2024-06-27

**Authors:** Guang Wang, Bin Chen, Xu Zhang, Guangzu Du, Guangyu Han, Jing Liu, Yuejin Peng

**Affiliations:** 1 Yunnan State Key Laboratory of Conservation and Utilization of Biological Resources, Yunnan Agricultural University, College of Plant Protection, Kunming, China; 2 Yunnan Key Laboratory of Potato Biology, School of Life Science, Yunnan Normal University, Kunming, China; University of Georgia, Athens, Georgia, USA

**Keywords:** bZIP-type transcription factors, yeast activator protein, *Beauveria bassiana*, virulence, oleic acid, humoral immunity

## Abstract

Basic leucine zipper domain transcription factors (TFs), of which yeast activator protein (Yap) is a significant class, are crucial for the development of sclerotia, the stress response, vegetative growth, and spore adhesion. Nevertheless, nothing is known about how Yap TFs contribute to the pathogenicity of entomopathogenic fungus. In this work, *Beauveria bassiana* was used to identify and knock out the yeast gene *BbYap1*, which is similar to Yap. The *BbYap1* gene deletion has an impact on lipid homeostasis of *B. bassiana*; oleic acid, for example, dropped by 95.69%. The *BbYap1* mutant exhibited much less virulence and vegetative development in comparison to the wild strain, while demonstrating a greater sensitivity to chemical stress. It is noteworthy that the physiological abnormalities brought on by *BbYap1* deletion were largely repaired by the addition of exogenous oleic acid, as seen by the notable increase in insect survival in the blood cavity injection group. Following infection with the *BbYap1* mutant, the host exhibits a considerable down-regulation of the expression of β-1,3-glucan recognition protein, gallerimycin, gloverin, and moricin-like protein genes. Likewise, the introduction of exogenous oleic acid markedly increased the host’s expression of the aforementioned genes. In summary, *BbYap1* regulates cellular enzyme lipid homeostasis and fungal virulence by eluding host humoral defense, which contributes to fungal chemical stress and vegetative development.

**IMPORTANCE:**

Entomopathogenic fungi (EPF) offer an effective and eco-friendly alternative to curb insect populations in biocontrol strategy. When EPF enter the hemolymph of their host, they encounter a variety of stress reactions, such as immunological and oxidative stress. Basic leucine zipper domain transcription factors, of which yeast activator protein (Yap) is a significant class, have diverse biological functions related to metabolism, development, reproduction, conidiation, stress responses, and pathogenicity. This study demonstrates that *BbYap1* of *Beauveria bassiana* regulates cellular enzyme lipid homeostasis and fungal virulence by eluding host humoral defense, which contributes to fungal chemical stress and vegetative development. These findings offer fresh perspectives for comprehending molecular roles of YAP in EPF.

## INTRODUCTION

A unique class of microorganisms known as entomopathogenic fungus (EPF) infects and kills insects and other arthropods (1). The attachment of conidia, germination, penetration, development, and production of secondary infectious conidia are the several categories of EPF parasitic processes (2). When fungi enter the hemolymph of their host, they form blastospores and hyphal bodies. Hyphal bodies encounter a variety of stress reactions, including immunological and oxidative stress, as they penetrate the host hemolymph (3–5). It is still unclear, therefore, how EPFs’ virulence and stress response are related.

Since all eukaryotic genomes contain a large family of dimerizing transcription factors (TFs), basic leucine zipper (bZIP) transcription factors are TFs that have been conserved throughout evolution (6). Two domains make up bZIP proteins: a highly conserved DNA-binding basic region and a varied leucine zipper (7). In pathogenic fungi, the bZIP-type TFs have diverse biological functions related to metabolism, development, reproduction, conidiation, stress responses, and pathogenicity (8–10). For *Metarhizium rileyi* (11), *Metarhizium robertsii* (12), and *Beauveria bassiana* (13), respectively, the function of bZIP-type TFs in EPF includes vegetative growth, conidia and microsclerotia formation, spore adherence, and virulence via the TF of activator protein 1, bZIP-type TF gene MAA_01736, and HapX. The first bZIP-type TF, yeast activator protein (Yap), was discovered in *Saccharomyces cerevisiae* (14). Subsequently, a subset of Yap TFs, Yap1–Yap8, were thoroughly characterized in yeast (15). Yap family bZIPs have been linked to the response to several forms of stress, such as oxidative, osmotic, pharmacological, nutritional, and iron stress (15–19). Remarkably, Yap1 of *Aspergillus fumigatus* was not a necessary component of virulence (20). Nevertheless, nothing is known about how Yap TFs contribute to the EPF’s pathogenicity. Examining the contributions and possible mechanisms of Yap TFs to the pathogenicity of the EPF is crucial.

The immunological response is an insect’s defense mechanism against potentially dangerous pathogenic pathogens. In insects, humoral immunity and cellular immunity make up their innate immune system (21). Defense molecule production is the primary means by which humoral immunity is maintained (22, 23). Hemocyte-mediated phagocytosis, encapsulation, and nodulation make up cellular immunity (24). EPF has developed a number of complex defense mechanisms against insect immunological responses in order to survive in the insect’s hemocoel (25, 26). By producing *Metarhizium* collagen-like protein, *Metarhizium anisopliae* blocked hemocytes from recognizing their hyphal bodies by masking antigenic structural components of the cell wall and providing hydrophilic negatively charged nature (27). *B. bassiana* controlled protease activity to prevent phenol oxidase activation (28). Nevertheless, no evidence has been found linking Yap TFs to the EPF control of host insect immunological responses. Notably, *BbOle1* gene in *B. bassiana* was targeted by HapX, a bZIP-type TF, which controlled the amount of oleic acid (OA) (13). According to recent investigations, human pathogenic fungi (*Pseudevernia furfuracea* and *Malassezia sympodialis*) can release OA and decrease the host’s immune response (29, 30). This suggests that OA can assist pathogenic fungi withstand the immune responses of host insects. It is currently unclear, nevertheless, if Yap of EPF affects the immunological responses of the host insect. The Yap homolog *BbYap1* of *B. bassiana* was used in this work.

Using the technique of *Agrobacterium*-mediated transformation, *BbYap1* was knocked out and was shown to be critical for *B. bassiana* pathogenicity, stress response, and radial growth. Interestingly, *BbYap1* mutant strain infection up-regulated most antimicrobial peptide-related genes and down-regulated most β-1,3-glucan recognition protein (βGRP)-related genes in *Galleria mellonella* hemocytes; these effects were reversed by exogenous OA.

## MATERIALS AND METHODS

### Strains and culture conditions

Fungal wild-type (WT) *B. bassiana* ARSEF2860 was kept on Sabouraud dextrose agar (SDAY; 1% peptone, 1% yeast extract, 4% glucose, and 1.5% agarose) at 25°C. For the purpose of creating the plasmid, *Escherichia coli* strain DH5*α* (Invitrogen, Carlsbad, CA, USA) was cultured at 28°C on Luria-Bertani medium and treated with the necessary antibiotic as a selection reagent. In the fungal transformation process, *Agrobacterium tumefaciens* AGL-1 was employed as a donor strain and was cultivated at 28°C in yeast extract media.

### Bioinformatics analysis and phylogenetic tree construction

BBA_04958’s associated sequences were retrieved from genomic database for *B. bassiana* ARSEF2860 (31). The conserved domain, which is the bZIP domain of Yap and related proteins, was found using the NCBI’s online Conserved Domain search (https://www.ncbi.nlm.nih.gov/Structure/cdd/wrpsb.cgi) (32). The NCBI database was used to download the homologs found in other fungi. Using 1,000 bootstrap replicates and the neighbor-joining method as its foundation, MEGA (version 7.0) created the phylogenetic tree. The web portal SMART (http://smart.embl-heidelberg.de/) identified the domains of these proteins (23). Next, IBS (Version 1.0: http://ibs.biocuckoo.org/online.php) was used to view the domains.

### Gene knockout and complementation

*BbYap1* disruption and complementation were carried out in the manner previously mentioned (33). The *BbYap1* open reading frame (ORF) sequences upstream (1 .13 kb) and downstream (1.47 kb) were amplified using the primer pairs P1/P2 and P3/P4 (Table S1), respectively, to create the gene disruption vector. Using the ClonExpress II one-step cloning kit (Vazyme Biotech, Nanjing, China), the upstream and downstream fragments were purified and separately ligated into the EcoRI/BamHI and XbaI sites of p0380-bar (providing resistance to ammonium glufosinate). The vector that emerged was called p0380-BbYap1-KO. The complemented strain was created by cloning the fragment with the native promoter (amplified using primer pair P7/P8; Table S1) and the complete ORF sequences of *BbYap1* into the vector p0380-sur-gateway, which confers resistance to chlorsulfuron. The resulting Δ*BbYap1* mutant strain was then transformed into the complemented strain.

Every fungal transformation experiment was carried out using techniques of *Agrobacterium*. Primers P5 and P6 were used to screen the potential mutants by PCR, and Southern blot analysis using a DIG DNA labeling and detection kit (Roche, Germany) was used to confirm the results. The probes were made using a fragment (319 bp) that was amplified using the primer pair P9/P10 (Table S1).

### Lipidomic analysis

The extraction of the lipids was performed as previously mentioned (13). To put it briefly, 100 mg of 7-day-old conidia on sabouraud dextrose agar with yeast extract medium (SDA) plates were suspended in 2.5 mL of water, and then 5.0 mL of chloroform and 2.5 mL of methanol were added. For 2 h, the ensuing suspension was maintained at −20°C. Subsequently, 2 mL methanol and 1 mL chloroform were added to the solution and mixed. The liquid’s bottom layer was taken into a tube and dried on a Termovap sample concentrator following a 1-h stratification period. After dissolving the sample in 1 mL of n-hexane and 0.25 mL of sodium methoxide (0.5 M), the methylation reaction was carried out for 30 min at 55°C. After being gathered, the n-hexane layer was dried. One hundred twenty microliters of n-hexane was used to dissolve the methylated fatty acids (FAs) and lipids thereafter. Following their separation using ultrahigh-performance liquid chromatography, these n-hexane solutions were examined using a Thermo Scientific Q Exactive Plus mass spectrometer. Lipid secondary identification and quantification were performed using Thermo Scientific Lipid Search v.4.1.30 software. An analysis of primary ion mass spectra was used to quantify the mass content of every identified molecule. The WT and mutant strains’ lipid molecules were evaluated for significant differences using variable importance in the projection (VIP) >1 and *P*-value <0.05. For every strain, there were three separate replicates.

### OA content assay

Using the previously mentioned techniques, total FAs were retrieved. A Focus series gas chromatograph (Thermo Scientific) connected to a DSQ2 mass selective detector (Thermo Scientific) was used to evaluate total fatty acid methyl esters (FAME). Peak regions were recorded using HP ChemStation software (version D.01.02.16, 2004) (13). Using Supelco 37 component FAME mixture as a standard (catalog no. 47885U, Sigma), FAME were detected and measured.

### Membrane integrity of fungal cells

SYTOX Green nucleic acid staining was used as previously described (13). Aliquots of a 100 µL conidial suspension (10^7^ conidia/mL) were cultured) on SDA plates for 12 h at 25°C. Cells were stained with 5 mM SYTOX Green for 10 min at 25°C in darkness. The fluorescent signals were observed under a laser scanning confocal microscope.

### Determination of biological phenotypes of fungi

Aliquots of wild-type, complementation, and disruption strains in 1 µL conidial suspension (10^6^ spore/mL) were inoculated onto SDAY and SDAY with 0.3% OA media, respectively. In a culture chamber, the plates were cultivated at 25 ± 1°C with a 12-h light/dark cycle. Conidia of WT, Δ*BbYap1*, and Δ*BbYap1* strains (1 µL of 10^6^ spore/mL) were inoculated into plates containing varying amounts of carbon (3% glucose and 3% trehalose) and nitrogen (0.3% NH_4_Cl, 0.3% GlcNAc, and 0.3% gelatin) in order to analyze the sources of carbon and nitrogen. Aliquots of 1 µL conidial suspension (10^6^ spore/mL) were spotted on SDAY media supplemented with 0.02% menadine and 0.5 M NaC for stress-resistant analysis. All plates were cultured for 7 days at 25°C under a 12:12-h light/dark cycle, and the colony’s growth status was recorded. As a measure of hyphal sensitivity to each stress, the percentage of relative growth inhibition was calculated using the formula (*d*_c_ − *d*_t_) / *d*_c_ × 100 (*d*_c_, control colony diameter; *d*_t_, stressed colony diameter).

### Insect bioassay

Cuticle infection and intrahemocoel injection tests were used to assess the impact of *BbYap1* deletion on conidial pathogenicity (34). *Galleria mellonella* larvae were inoculated into the insects for the cuticle infect assay by submerging them in the conidial suspension (10^7^ conidia/mL) for a duration of 15 seconds. Aliquots of a 5 µL conidial suspension (10^5^ conidia/mL) were injected into *G. mellonella* hemocoel for the intrahemocoel injection test. Every bioassay experiment was run in three parallel duplicates, with each replicate containing roughly 30 to 35 larvae. The median lethal time (LT_50_) was computed using daily survival data collection.

Conidial suspension (10^5^ conidia/mL; 5 µL aliquots) was injected into the host hemocoel to monitor the formation of *in vivo* hyphal structures. At 12, 24, 36, 48, 60, 72, and 84 h post-injection (HPI), larvae that had received conidia injections were killed, and the hemolymph was diluted 1:1 in sterile anticoagulant (0.14 M NaCl, 0.1 M glucose, 25 mM sodium citrate, and 30 mM citric acid). Hyphal bodies were examined under a microscope and captured on camera.

### Measurement of phenoloxidase activity

An insect hemolymph’s phenoloxidase (PO) activity was measured using a PO kit from the Jiancheng Bioengineering Institute in Nanjing, China. Abdominal tarsi of *G. mellonella* larvae had been cut at 48 h post-infection, the hemolymph was extracted and diluted 1:3 in sterile anticoagulant buffer. Following centrifugation of the hemolymph-anticoagulant combination at 94 g for min at 4°C, plasma (cell-free) was then recovered (35). The supernatant was used in accordance with the manufacturer’s instructions for PO determination using the PPO kit. The hemocyte precipitate was utilized to extract total RNA.

### Quantitative real-time reverse-transcription polymerase chain reaction (RT-qPCR)

In accordance with the manufacturer’s instructions, total RNA was obtained using the PastPure Cell/Tissue Total RNA Isolation Kit-BOX1 (Vazyme, Nanjing, China). Using HiScript III All-in-one RT SuperMix Perfect for qPCR (Vazyme, Nanjing, China) and the manufacturer’s instructions, cDNA was made from whole RNA. To standardize the quantity of RNA template, the 18S rRNA gene was employed as an internal reference. The 2^-ΔΔCT^ technique was utilized to determine the relative expression of every gene (36). Table S2 contains a list of primers utilized in RT-qPCR tests.

### Statistical analysis

Tukey’s honestly significant difference test was used to determine the significance of the indicated phenotypes among different strains.

## RESULTS

### Sequences analysis of Yap1 in *B. bassiana*

**Fig 1:**
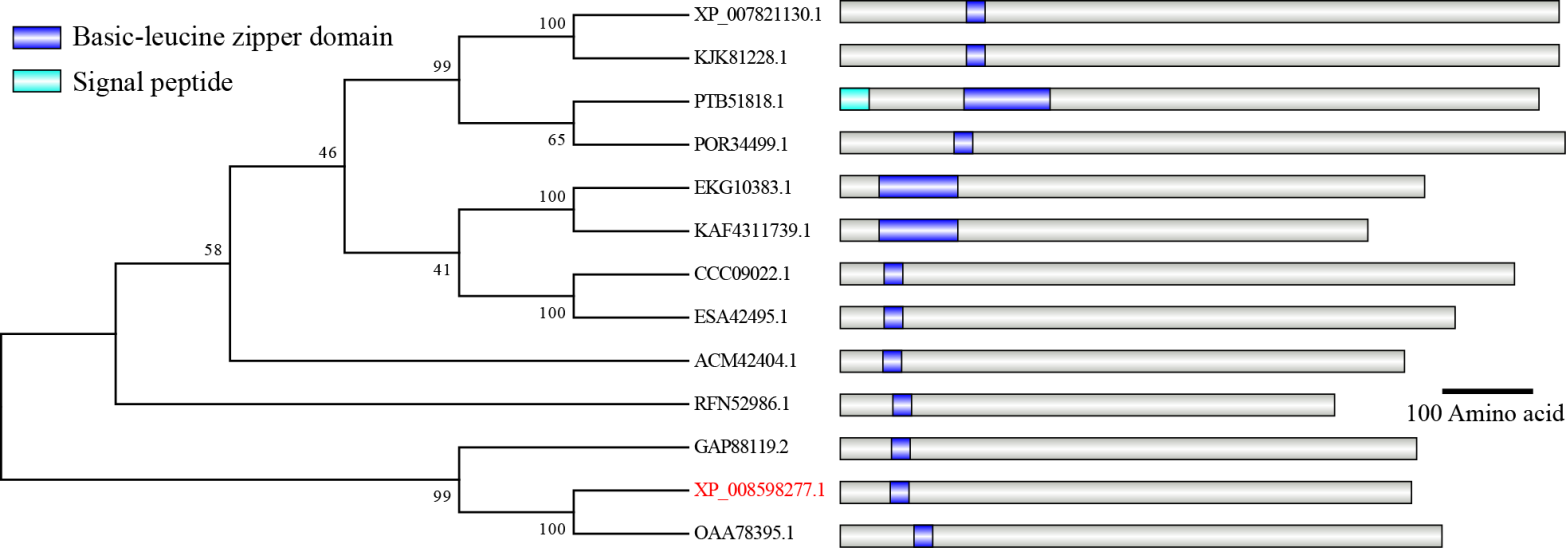
Phylogenetic analysis and sequence comparison of BbYap1 protein and its orthologs. The phylogenetic tree was made by MEGA based on neighbor-joining method method with 1,000 bootstrap replicates. The sequences of XP_007821130.1 (*Metarhizium robertsii*), KJK81228.1 (*Metarhizium anisopliae*), PTB51818.1 (*Trichoderma harzianum*), POR34499.1 (*Tolypocladium paradoxum*), EKG10383.1 (*Macrophomina phaseolina*), KAF4311739.1 (*Botryosphaeria dothidea*), CCC09022.1 (*Sordaria macrospora*), ESA42495.1 (*Neurospora crassa*), ACM42404.1 (*Floropilus chiversii*), RFN52986.1 (*Fusarium flagelliforme*), GAP88119.2 (*Rosellinia necatrix*), XP_008598277.1 (*Beauveria bassiana*), and OAA78395.1 (*Akanthomyces lecanii*) were downloaded from NCBI.

Through homologous recombination, the *BbYap1* target deletion mutant was created in *B. bassiana* to determine the possible function of BbYap1 (Fig. 1). The Δ*BbYap1* strain was then ectopically integrated with the full *BbYap1* gene to reverse the effects of gene loss (Fig. S1A). Transformants were confirmed by using PCR. As shown in Fig. S1B, a 527 bp DNA fragment was amplified from the WT and Δ*BbYap1::BbYap1* strains, but Δ*BbYap1* strain did not. The Δ*BbYap1* and Δ*BbYap1::BbYap1* strains amplified a 1,114 bp fragment by PCR, while the WT strain did not. Southern blotting was used to confirm the existence of Δ*BbYap1* and Δ*BbYap1::BbYap1* strains (Fig. S1C). These findings proved that *BbYap1* disruption and complementation were built successfully

### Roles of *BbYap1* in radial growth and stress response of *B. bassiana*

**Fig 2:**
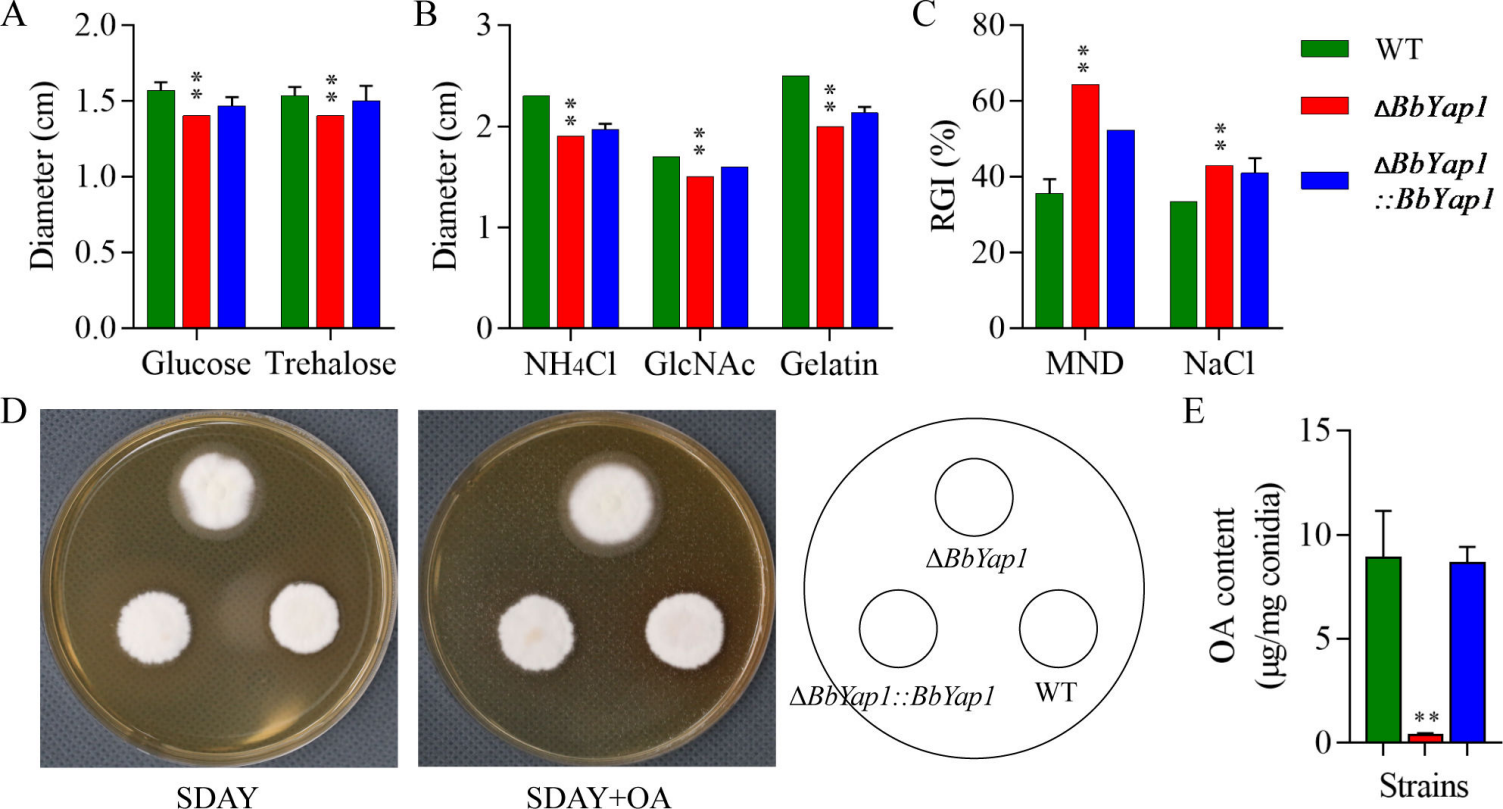
Roles of *BbYap1* in radial growth and stress response of *B. bassiana*. Phenotypic comparison between the WT and mutant strains on the carbon (**A**) and nitrogen (**B**) sources. (**C**) Phenotypic comparison between the WT and mutant strains on the chemical stress. (**D**) The effect of exogenous OA on radial growth. (**E**) *BbYap1* mutation reduced OA content of *B. bassiana* conidia. *P* < 0.05 (*) and *P* < 0.01 (**).

Δ*BbYap1* strain growth defect on glucose and trehalose (Fig. 2A) and NH_4_Cl, GlcNAc, and gelatin (Fig. 2B). The Δ*BbYap1* strain demonstrated a considerable increase in sensitivity to both osmotic stresses caused by NaCl and oxidative stress created by menadione when compared to the WT strain (Fig. 2C). Furthermore, on SDAY plates, the disruption mutant showed a small growth deficit that was corrected by exogenous OA (Fig. 2D). Gas chromatography mass spectrometry (GC-MS) results showed that the absence of *BbYap1* resulted in a significant decrease in OA content in fungal spores (Fig. 2E). Notably, OA deprivation resulted in poor membrane integrity in *B. bassiana* in germlings (13). As shown in Fig. S2, loss of *BbYap1* led to poor membrane integrity in germlings. According to these findings, *BbYap1* supports the radial development and stress response of *B. bassiana*.

### *BbYap1* contributes to the virulence of *B. bassiana*

**Fig 3:**
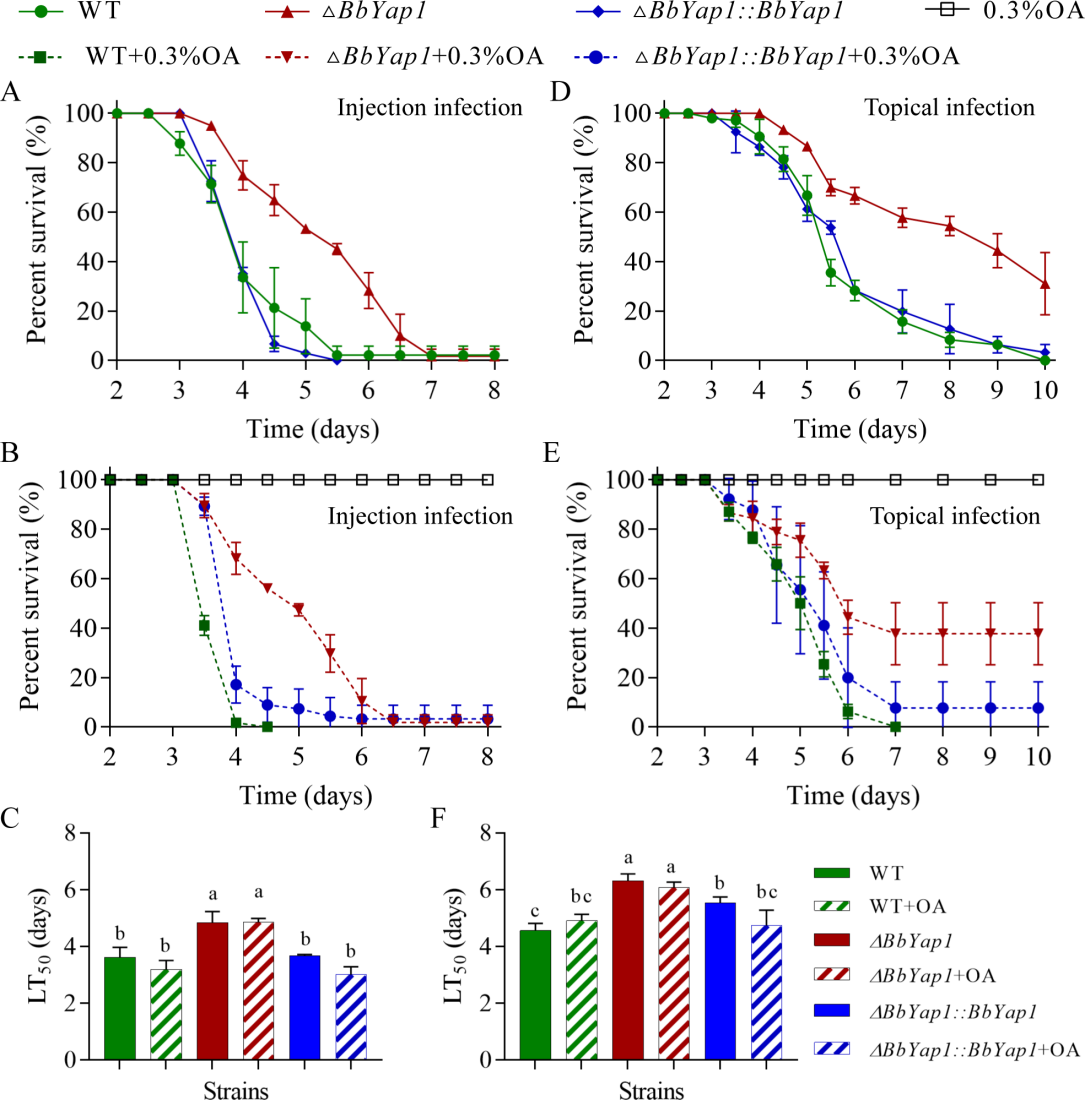
*BbYap1* contributes to the virulence of *B. bassiana*. Conidial virulence was evaluated with injection infection (**A**) and topical infection (**D**) bioassays. The effect of exogenous OA on conidial virulence with injection infection (**B**) and topical infection (**E**) bioassays. The LT_50_ was measured by Kaplan-Meier method in the injection infection (**C**) and topical infection (**F**) bioassays. The letter represents a significant difference at the level *P* < 0.05.

The survival rate of insects was assessed to determine how well *BbYap1* functioned in the pathogenicity of *B. bassiana*. In contrast to the larvae treated with WT and mutation strains, which reached survival rates of 2.22% and 43.33% at 5.5 days after injection treatment, the complementation strain-treated larvae died completely (Fig. 3A). The pathogenicity of *B. bassiana* was also used to investigate the role of OA. The strains of WT, Δ*BbYap1*, and Δ*BbYap1::BbYap1* had LT_50_ of 3.62, 4.84, and 3.69 days, respectively (Fig. 3C). The larvae treated with WT strain died entirely by day 4.5 after exogenous addition of OA, but the larvae treated with complementation and mutation strains survived at rates of 8.97% and 56.15%, respectively (Fig. 3B). Depending on the group, the LT_50_ values were 3.19, 4.87, and 3.01 days for WT + OA, Δ*BbYap1* + OA, and Δ*BbYap1::BbYap1* + OA (Fig. 3C). The *ΔBbYap1* strain-infected insects showed extremely high survival (31.11%) at 10 days post-infection in the cuticle infection, while the survival rates of the WT and complementation strains were 0% and 3.33%, respectively (Fig. 3D). According to Fig. 3F, the LT_50_ values for the WT, Δ*BbYap1*, and Δ*BbYap1::BbYap1* strains were 4.57, 6.32, and 6.03 days, respectively. The larvae treated with the WT strain died entirely after the exogenous injection of OA on day 7, but the larvae treated with the complementation and mutation strains survived at rates of 42.03% and 7.78%, respectively (Fig. 3E). As shown in Fig. 3F, the LT_50_ for the WT + OA, Δ*BbYap1* + OA, and Δ*BbYap1::BbYap1* + OA groups were 4.92, 6.08, and 4.74 days, respectively. These findings proved that *BbYap1* is essential to fungal virulence, and exogenous OA are unable to make up for *BbYap1* loss.

### *BbYap1* regulates lipid homeostasis

**Fig 4:**
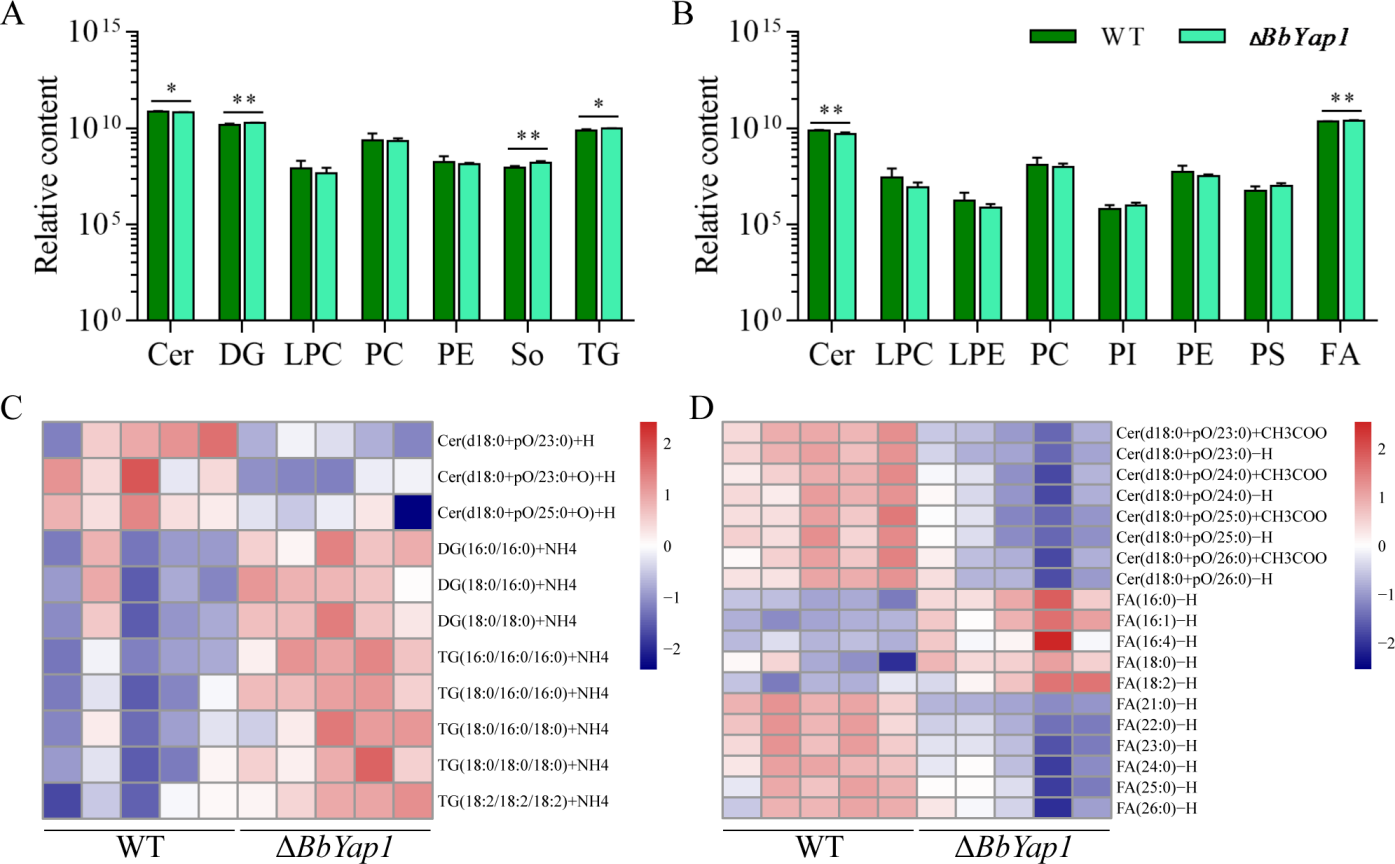
*BbYap1* regulates lipid homeostasis. The relative content of lipid class in the cationic (**A**) and anionic (**B**) profiles. Heatmap showed significant differences of lipids in the cationic (**C**) and anionic (**D**) forms, respectively. *P* < 0.05 (*) and *P* < 0.01 (**). LPC, lysophosphatidylcholine; PC, phosphatidylcholine; PI, phosphatidylinositol; PS, phosphatidylserine.

In the conidia of *B. bassiana*, we previously discovered that the bZIP-type TF HapX controlled phospholipid homeostasis. Lipidomic analysis was used to assess the lipid composition of conidia. Lipid molecules exist in both cationic and anion forms (Table S3A and B). The *BbYap1* mutant strain exhibited a considerable drop in the lipid class of ceramides (Cer) in the cationic profiles (Fig. 4A) as compared to the WT strain. The lipid classes of diglyceride (DG), sphingosine (So), and triglyceride (TG) were all markedly elevated by the *BbYap1* mutant. The *BbYap1* mutant dramatically boosted and decreased the FA lipid class and the Cer lipid class in the anionic profiles (Fig. 4B), respectively. Based on variations in the length or unsaturation of the carbon chain, each class of lipids is further classified into distinct molecular species. Three Cer species were considerably fewer in the Δ*BbYap1* strain’s cationic profiles (Fig. 4C) when compared to the WT strain. When comparing the Δ*BbYap1* strain to the WT strain, there was a significant increase in three DG and five TG species (Fig. 4C). The *BbYap1* mutant drastically reduced eight Cer species in the anionic profiles (Fig. 4D). Five and six FA species, respectively, were greatly enhanced and lowered by the *BbYap1* mutant (Fig. 4D). Notably, OA belongs to the category of FA; GC-MS results showed that *BbYap1* mutant significantly decreased OA content (Fig. 2E). These findings suggested that *BbYap1* is essential to lipid homeostasis in *B. bassiana* conidia.

### Exogenous OA reverses the immune response of *G. mellonella* hemocytes to *BbYap1* mutant strain

**Fig 5:**
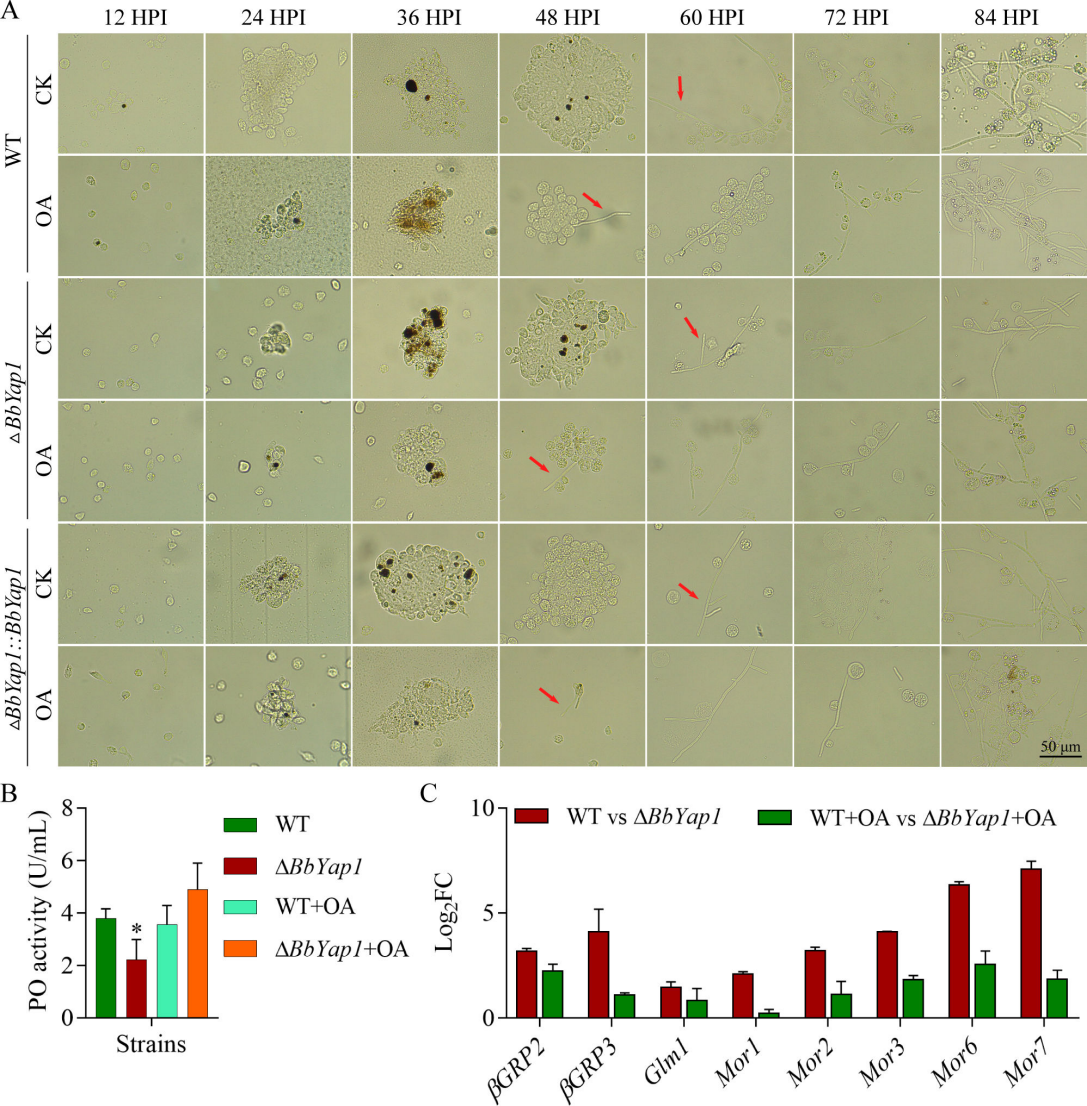
Exogenous OA regulates the immune response of *G. mellonella* hemocytes to *BbYap1* mutant strain. (**A**) Conidial suspension (5 µL of 10^5^ conidia/mL) was injected into host and incubated at 25℃. The hemocyte encapsulation was examined under a fluorescence microscope at an interval of 12 h. Scale bars = 50 µm. (**B**) The activity of PO was measured in the intrahemocoel injection treatment with exogenous oleic acid. (C) β-1,3-glucan recognition protein and antimicrobial peptide-related genes expression were measured at 24 HPI. *P* < 0.05 (*). Glm1, gallerimycin; Glv, gloverin; Mor, moricin-like protein.

In comparison to the WT and complementation strains, the mutant strain showed delayed LT_50_ values at conidia-infected or conidia-injected conditions; the delay in LT_50_ values was greater in the conidia-injected treatment (Fig. 3). In order to observe fungal colonization in the host body, we conducted an additional experiment using a conidial concentration of 10^5^ conidia/mL. Following conidia injection, host hemocytes gathered and created melancholic dots (Fig. 5A). Up to 48 HPI, there were no appreciable variations in the hemocyte response between the WT, Δ*BbYap1*, and Δ*BbYap1::BbYap1* strains. All strains started to generate hyphal bodies from the host hemocoel at 60 HPI. Numerous free-floating hyphal entities were formed at 72 HPI by WT, mutation, and complementation strains (Fig. 5A). The degree of humoral immunity to plasma PO in *G. mellonella* was measured. At 48 HPI, *BbYap1* deletion exhibited reduced plasma PO activity in comparison to the WT strain (Fig. 5B). Infection with the Δ*BbYap1* strain decreased PO activity when exogenous OA was applied. Next, we observed the immunity-related genes’ transcriptional responses at 24 HPI (Fig. 5C). When compared to the WT strain, the expression of the βGRP-related genes *βGRP2* and *3* was reduced by *BbYap1* deletion, but this effect was restored by exogenous OA. The Δ*BbYap1* strain infection decreased the expression of the gallerimycin gene *Glm1* and the genes encoding moricin-like proteins (*Mor1*, *2*, *4*, *6*, and *7*). This was reversed by exogenous OA. When combined, these findings suggested that exogenous OA reverses the host’s immunological response, which is influenced by *BbYap1*.

## DISCUSSION

In a class of fungi, yeast activator protein is a significant alkaline leucine zipper transcription factor that is involved in spore adhesion, vegetative growth, and stress response (14). In this work, we offer the first proof that BbYap1 controls lipid homeostasis to aid in host cell defense and immunization against entomopathogenic fungus. Plants’ capacity to withstand environmental stress is greatly aided by bZIP-type TFs (37). bZIP-type TFs are linked to lipid metabolism in a variety of taxa, including microbes, plants, and mammals, in addition to controlling environmental stress (7). In plants, the lipid productivity in the *Nannochloropsis salina* was increased by over-expression of ZIP1 (bZIP-type TF) (38, 39). The production of total lipids and docosahexaenoic acid in fungus was enhanced by 30.1% and 46.5%, respectively, upon deletion of the fabR gene (bZIP-type TF) in *Schizochytrium* sp. (40). Four fatty acid concentration in *B. bassiana* was significantly reduced as a result of ablation of *BbHapX* (bZIP-type TF) (13). Additionally, our findings demonstrated that *BbYap1*, a mutation of the bZIP transcription factor, reduced lipid homeostasis and membrane integrity in *B. bassiana* conidia and a noteworthy 95.69% reduction in OA content. It’s interesting to note that *BbHapX* disruption resulted in poor membrane integrity in *B. bassiana* in *in vivo* blastospores and germlings (13). Membrane integrity is disrupted when endogenous- or exogenous-free (unesterified) FAs build up in the cell (41). Our findings lend credence to the hypothesis that *BbYap1* plays a significant role in maintaining cell membrane homeostasis and lipid homeostasis, which are essential for the pathogenicity of filamentous fungus (42, 43). Here, we discovered that the *BbYap1* mutant significantly decreased pathogenicity of *B. bassiana* conidia, indicating that *BbYap1* knockdown impacted pathogenicity through controlling membrane integrity and fluidity.

Large-scale genome expression reprogramming driven by many TFs is required for yeast adaptation to stress (44). A number of fungi species, including fungi like *S. cerevisiae*, *B. bassiana*, and *Aspergillus nidulans* are subject to biotic and abiotic stressors due to the involvement of certain bZIP transcription factors (44–47). BbYap1 in this study belonged to the superfamily of bZIP transcription factors that resemble activator protein-1 (AP-1). Significantly more vulnerable to chemical and biological stress was *BbYap1* knockdown. The yeast AP-1-like TFs play an important role in controlling oxidative stress, cadmium, osmotic shock, iron overload, hydroquinone, and nitrosative stressors (18, 44) , which implies that BbYap1 is essential for controlling B. bassiana adapts to stress. In order to generate hyphal bodies and blastospores, entomopathogenic fungi need to overcome oxidative stress in the host hemolymph (48). Significantly less blastospore formation and hyphal body expansion were seen in the host hemocoel of the *BbHapX* mutant strain (13). In line with earlier findings, we discovered that the *BbYap1* mutant strain considerably decreased the generation of plasma blastospores and showed a minor growth deficiency on SDAY plates. According to this study, *BbYap1* might help prevent oxidative stress in *B. bassiana* cells.

bZIP-type TF knockdown decreased the virulence of entomopathogenic fungus *M. robertsii* (12), *M. rileyi* (11), as well as *B. bassiana* (13). We saw that the pathogenicity of *B. bassiana* was diminished by *BbYap1* deletion. Blastospores, which are known as a covert growth form of fungi to circumvent host immunity and aid in conquering the host by creating toxic secondary metabolites, are known to play a significant role in the pathogenicity of fungi throughout the development of disease (49). Our findings also demonstrated a considerable reduction in blastospore count and PO activity following *BbYap1* knockdown. A crucial enzyme in the process of melanization, PO is inhibited by serine protein inhibitors and activated by serine proteins (50). By producing oosporein, fungi prevent PO from being activated (51). In light of the findings of our investigation, we hypothesize that *BbYap1* contributes to the pathogenicity of *B. bassiana* by controlling host defenses.

The fluidity of the membrane is mostly dependent on OA (52). According to a prior study, *BbHapX* compromised the membrane integrity of *B. bassiana* conidia by providing OA (13). Here, we discovered that the *BbYap1* mutant significantly decreased the OA content of *B. bassiana* conidia, indicating that *BbYap1* knockdown altered membrane fluidity through controlling the metabolism of OA. According to a recent study, OA increased conidial production and quality in *M. rileyi* and *B. bassiana* conidia (42, 53). Furthermore, OA promotes G-protein coupled receptors membrane docking and activity as well as related signaling molecules (52). For example, OA increased the expression of growth and development-related genes (*Mrap1*, *MrNsdD*, *MrPbs2*, *MrSwi6*, *MrSte12*, and *MrMsn2*) in *M. rileyi* (53), suggesting that OA functions as a signaling molecule in the production of fungal conidia. Unsaturated fatty acids, or OAs, are involved in immunological responses (54, 55). In this investigation, we discovered that *BbYap1* deletion decreased the OA content in *B. bassiana* conidia. The down-regulation of most genes associated to β-1,3-glucan recognition protein, in *G. mellonella*, was reversed by exogenous OA and hemocytes and the majority of antimicrobial peptide-related genes being up-regulated as a result of infection with the *BbYap1* mutant strain. The genes linked to immunity, namely β-1,3-glucan recognition protein, and antimicrobial peptide suggest that *BbYap1* modulates the host’s immunological response through controlling the amount of OA present. However, we are still unclear that OA directly affects host immune reaction or indirectly functions by affecting fungal lipid metabolism, which should be investigated in the future.

Our investigation demonstrates that *BbYap1* adds to the pathogenicity of *B. bassiana* and is mostly dependent on the host hemolymph’s fast multiplication, which could be brought on by the gene’s involvement in processes such as fungal host immunity evasion. Fungal spore lipid homeostasis was impacted by *BbYap1* loss, with a notable drop in OA content. Exogenous OA makes up for the immunological and physiological deficits brought on by this gene’s loss. These findings offer fresh perspectives on the molecular role of *Yap1* in EPF.

## Data Availability

The data that support the findings of this study are available from the corresponding author upon reasonable request. The data that support the findings of this study are openly available in figshare at http://doi.org/10.6084/m9.figshare.25600203

## SUPPLEMENTAL MATERIAL

Supplemental material msphere.00351-24-s0001.docx Figures S1 and S2; Tables S1 and S2.

Table S3 msphere.00351-24-s0002.xlsx Effect of BbYap1 on lipid content in conidia of *B. bassiana*.

ASM does not own the copyrights to Supplemental Material that may be linked to, or accessed through, an article. The authors have granted ASM a non-exclusive, world-wide license to publish the Supplemental Material files. Please contact the corresponding author directly for reuse.
