# Supplementary material for: The basic leucine zipper domain (bZIP) transcription factor BbYap1 promotes evasion of host humoral immunity and regulates lipid homeostasis contributing to fungal virulence in Beauveria bassiana
Source: mSphere. 2024 Jun 27;9(7):e00351-24. doi: 10.1128/msphere.00351-24 (PMC11288043; doi:10.1128/msphere.00351-24)
Supplement: Supplemental material — Figures S1 and S2; Tables S1 and S2. [file msphere.00351-24-s0001.docx]

**Supplementary Materials**


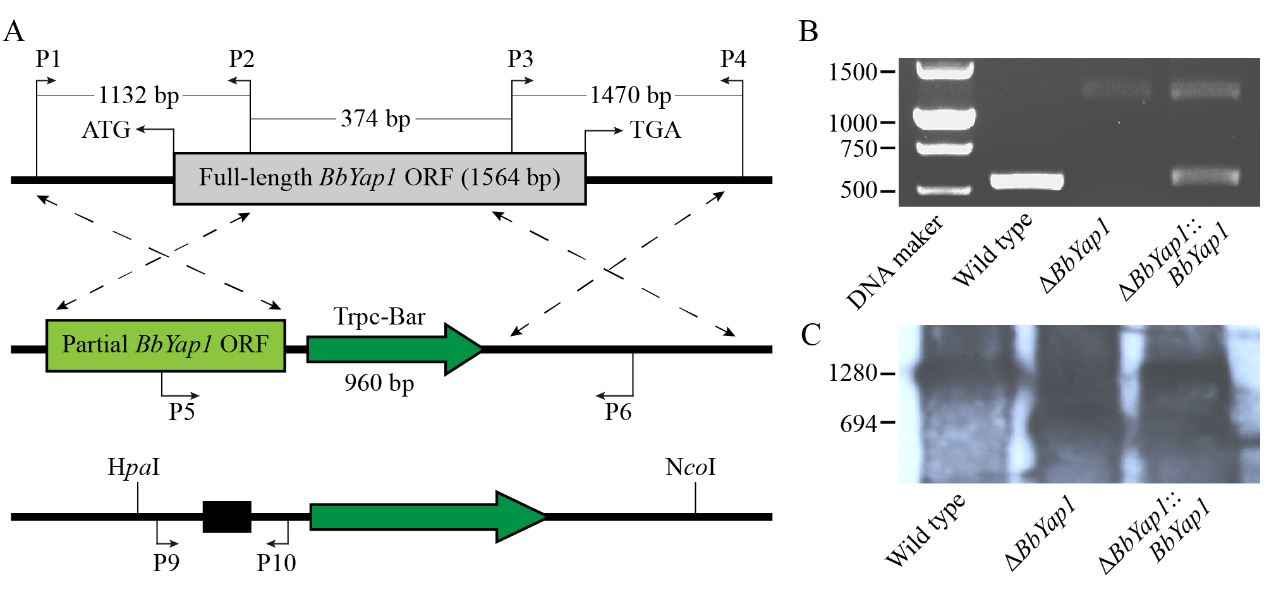


**Figure S1. Molecular manipulation for gene function analysis of *B. bassiana*.** (**A**) Schematic diagram of gene disruption in fungal genome using homologous recombination strategy. Candidate transformants were screened by PCR (**B**) and further confirmed by Southern blot analysis (**C**). In Southern blotting, the enzyme pair SacⅠ/NcoⅠ was used to digest genomic DNA. The numbers near the images in (**B**) and (**C**) indicate the molecular sizes of DNA fragments.


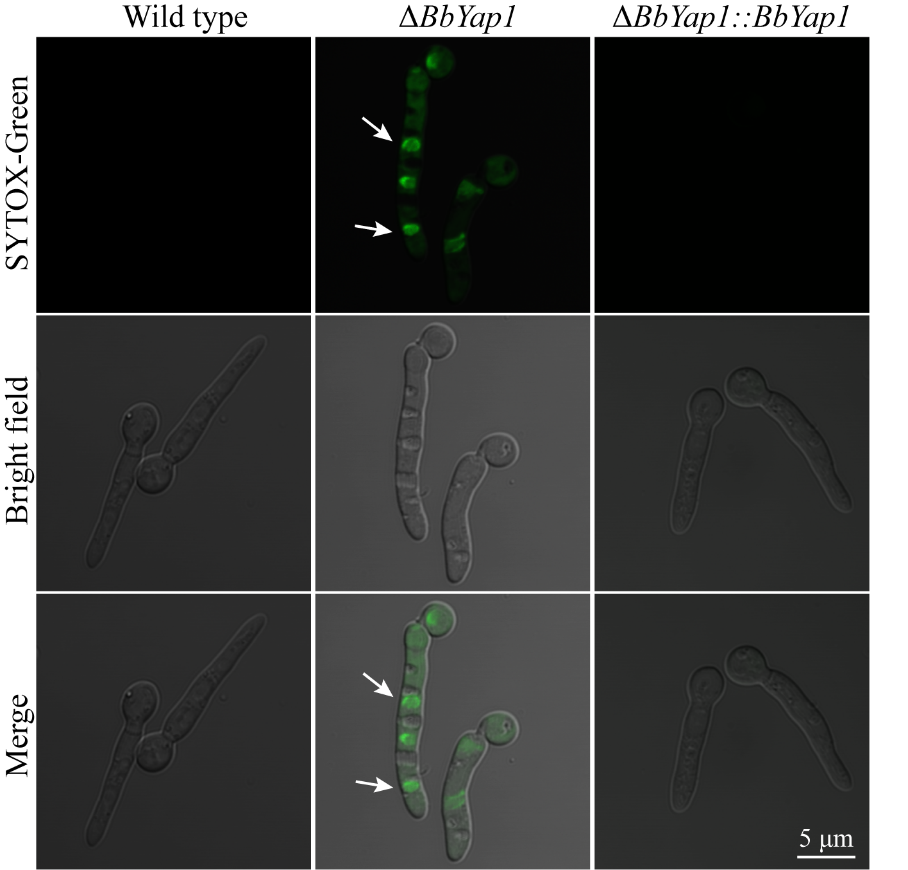


**Figure S2. SYTOX Green staining of *B. bassiana* germlings.** The stained nuclei (green) in the fungal cells are indicated with white arrows.

**Supplementary Table 1. Primers used for molecular manipulation in this study.**

| Primer  name | Sequence (5'-3') | Purpose of use |
| --- | --- | --- |
| P_1_ | **TGGGCCCGGCGCGCCGAATTC**TCCGAGACAGTCATCATTCA | Obtaining upstream flanking sequence |
| P_2_ | **TGGCTGCAGGTCGACGGATCC**GGAACCAGGACAAGAGGAAC |  |
| P_3_ | **GACCCATGGCTCGAGTCTAGA**TGCCCAGAGAACATACCG | Obtaining downstream flanking sequence |
| P_4_ | **GGTGGTGGTGGCTAGCGTCTAGA**ACAAGACGAGCCCAACGA |  |
| P_5_ | CAACTCCCTCGTCCTGGC | Confirming the candidate transformants |
| P_6_ | GGTTGCCTGTCGTTTCGT |  |
| P_7_ | **GGGGACCACTTTGTACAAGAAAGCTGGGT**TTGTCAACCATCGTGCTCTTG | Amplifying *HapX* for complementation |
| P_8_ | **GGGGACAAGTTTGTACAAAAAAGCAGGC**GCTTGCCAGTCACTACCTTG |  |
| P_9_ |  | Probe preparing in Sourthern blot |
| P_10_ |  |  |

Note: The underlined sequences in primers are required for cloning the PCR products into plasmids by homologous recombination.

**Supplementary Table 2.** RT-qPCR primers used in this study.

| Genes (Accession number) | Forward (5’-3’) | Reverse (5’-3’) |
| --- | --- | --- |
| *βGRP2* (LOC113513109) | ATTACAGATGGCATATCC | CTTGGACATTCTCTTTCT |
| *βGRP3* (LOC113521212) | TTAGAGACAGGAAGATTC | GAACATATTGGAGTATTATT |
| *Glm1* (LOC113523440) | TACAGAATCACACGACAT | TTGTAACCTCTACTCCTG |
| *Mor1* (LOC113509608) | CTCTTTATAGGGTCAAATGA | GCTGCACTGATTACTTTA |
| *Mor2* (LOC113509615) | ATGATGGTGATGGCTATG | TATTGATTCCACGCAGAG |
| *Mor3* (LOC113509614) | TGCTCGCCCTGTTTGTTG | CACCAATTACACCAAGACCTTT |
| *Mor6* (LOC113509609) | CGGTTTATTCCTTATGATTATG | TATAGCTTTACCACCCTTT |
| *Mor7* (LOC113509611) | AGTTCTTCAATCTCGTAT | AATAATTTTTCCGCCTTT |
| *18S rRNA* (LOC113514995) | AACCTGTTAAGAGACTGTAT | GCCTGTGTAGTGAGTAAT |

Note: βGRP, β-1,3-glucan-recognition protein; Glm1, Gallerimycin; Mor, Moricin-like protein; 18S rRNA, internal reference gene.
